# Geometric morphometrics defines shape differences in the cortical area map of C57BL/6J and DBA/2J inbred mice

**DOI:** 10.1186/1471-2202-7-63

**Published:** 2006-09-15

**Authors:** David C Airey, Fangbai Wu, Monica Guan, Christine E Collins

**Affiliations:** 1 Department of Pharmacology, Vanderbilt University Medical Center, Nashville, TN, USA; 2 Department of Psychology, Vanderbilt University, Nashville, TN, USA

## Abstract

**Background:**

We previously described planar areal differences in adult mouse visual, somatosensory, and neocortex that collectively discriminated C57BL/6J and DBA/2J inbred strain identity. Here we use a novel application of established methods of two-dimensional geometric morphometrics to examine shape differences in the cortical area maps of these inbred strains.

**Results:**

We used Procrustes superimposition to align a reliable set of landmarks in the plane of the cortical sheet from tangential sections stained for the cytochrome oxidase enzyme. Procrustes superimposition translates landmark configurations to a common origin, scales them to a common size, and rotates them to minimize an estimate of error. Remaining variation represents shape differences. We compared the variation in shape between C57BL/6J and DBA/2J relative to that within each strain using a permutation test of Goodall's F statistic. Significant differences in shape in the posterior medial barrel subfield (PMBSF), as well as differences in shape across primary sensory areas, characterize the cortical area maps of these common inbred, isogenic strains.

**Conclusion:**

C57BL/6J and DBA/2J have markedly different cortical area maps, in both size and shape. These differences suggest polymorphism in genetic factors underlying cortical specification, even between common isogenic strains. Comparing cortical phenotypes between normally varying inbred mice or between genetically modified mice can identify genetic contributions to cortical specification. Geometric morphometric analysis of shape represents an additional quantitative tool for the study of cortical development, regardless of whether it is studied from phenotype to gene or gene to phenotype.

## Background

**Figure 1:**
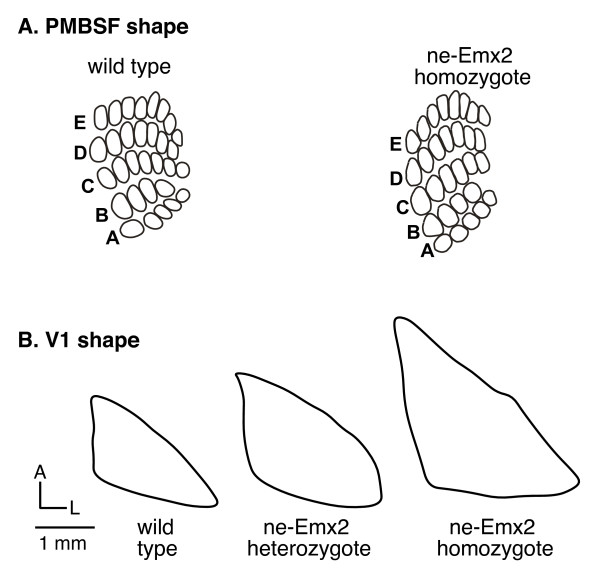
**Emx2 cortical area map phenotypes**. Outline drawings of cortical map phenotypes in wild type mice and mice over expressing *Emx2 *show changes in the shape of PMBSF and V1. 1A shows PMBSF for wild-type and *ne-Emx2 *homozygote. 1B shows V1 drawings from wild-type, *ne-Emx2 *heterozygote, and *ne-Emx2 *homozygote. Rostral is up, lateral is right. In both areas, increasing *Emx2 *expression produces rostral elongation, although in PMBSF the lateral edge appears more affected than the medial edge. Drawings have been adapted from [5] with permission from Dr. D. D. O'Leary.

Experimental genetic manipulations have produced spectacular alterations in the position, size, and shape of primary sensory and motor areas of the mouse neocortex, leading to the conclusion that cortical arealization results in part from intrinsic cortical genetic mechanisms acting early in development [1-4]. For example, Hamasaki et al., [5], manipulated expression of the gene *Emx2 *by transgenic methods. With increasing *Emx2 *expression, caudal cortical areas increased in size and rostral extent, whereas more rostral areas shifted position and decreased in size. Figure 1 shows the outlines of primary visual cortex (V1) and the posterior medial barrel subfield (PMBSF) in primary somatosensory cortex (S1) from representative wild-type and *ne-Emx2 *mice (adapted from [5] with permission). As a proportion of the total cortical sheet, the area of V1 was 52% increased and the area of PMBSF was 25% decreased in *ne-Emx2 *mice compared to wild-type mice [5]. Motor cortex, the most rostral cortical area measured, showed a 36% decrease in *ne-Emx2 *mice. In addition to alteration of cortical area size, cortical area shape changes are evident in Figure 1. The shapes of both V1 and the PMBSF are increased in rostral extent in *ne-Emx2 *mice, although the changes in PMBSF shape are also more complex.

Similar differences may be present between standard inbred strains of mice. Such differences, although likely more subtle than those induced by Hamasaki and colleagues, suggest polymorphism in genes underlying morphogenesis of the cerebral cortex. Because of the extensive changes in the cortical area map of transgenic *Emx2 *mice, the quantitative descriptions used by Hamasaki et al. were adequate in portraying the visible differences in area and shape [5]. When differences in the cortical area map are more subtle, then more sensitive, multivariate statistical methods are required. We previously demonstrated significant differences in the size of both V1 (12%) and the PMBSF (10%) between the inbred strains C57BL/6J and DBA/2J [6]. A multivariate logit model predicted strain identity with 90% correct classification rate [6]. We were unable, however, to demonstrate any reliable differences in landmark positions in the cortical area map using the methods described by Hamasaki et al., [5]. In this paper, we use established statistical methods to analyze shape, called geometric morphometrics (see [7-9] for introductions to shape analysis, and Methods for more details).

The analysis of shape has been successfully used to investigate neuroanatomical differences, but mostly within the context of human brain imaging data. For example, using anatomical landmarks from two dimensional magnetic resonance image (MRI) scans of the human midsagittal plane, Gharaibeh et al., [10], examined shape changes in the configuration of landmarks following the first episode of schizophrenia. Bookstein et al., [11], used other geometric morphometric methods to describe shape differences in the corpus callosum of fetal alcohol affected human brains, again from MRI scans. Computational anatomy methods have also been developed to describe shape changes in three dimensional data (e.g., [12,13]). The lissencephalic mouse cortex, in stained tangential sections, lends itself well to the application of established geometric morphometric methods for landmarks in two dimensions. There is a long history of using tangential cortical tissue sections in neuroanatomy, but the application of geometric morphometric methods to this histological preparation is novel.

**Figure 2:**
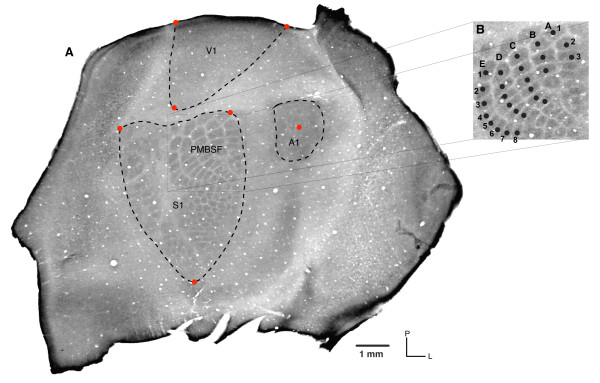
**Cortical landmarks**. (2A) A cytochrome oxidase stained tangential cortex section with locations of landmarks indicated in V1, S1, and A1. For V1 and S1, medial-, lateral-, and rostral-most corners were used. For A1, the centroid of the bounded region was used. (2B) For the PMBSF (inset), the centroid of each drawn barrel was used. Rostral is down, lateral is right.

When a tangential section of the adult mouse cortex is stained for the cytochrome oxidase enzyme, the outlines and components of primary visual, somatosensory (including PMBSF), and auditory cortex can be reliably discerned (Figure 2). The rodent somatosensory barrel field has been subjected to a number of quantitative analyses under a range of conditions, because barrels provide natural landmarks (for example, [14-17]). Each barrel in the PMBSF represents a primary facial vibrissa, and the arrangement of barrels in the cortex preserves the spatial arrangement of whiskers in the periphery. The regular topology of the PMBSF suggests it may serve as a useful indicator of shape changes in the cortical area map. Other landmarks may help assess more directly shape change across the entire cortical sheet.

Using two sets of landmark configurations, one local (PMBSF) and one more global (V1, S1, A1), we sought to identify differences in shape of primary sensory cortex between C57BL/6J and DBA/2J inbred strains. We collected landmark data from cytochrome oxidase stained sections of flattened cortex and compared landmark configurations using the Procrustes transformation, a standard method in the analysis of shape (see Methods). Landmarks included the positions of barrels in the PMBSF and minimal landmarks for the triangular shapes of primary visual cortex (V1) and primary somatosensory cortex (S1), and the position of the centroid of primary auditory cortex (A1) (see Figure 2). A single landmark for A1 is used, because A1 is ovoid in shape and lacks reliable outline landmarks, as in V1 or S1. Similarly, centroids were used as landmarks for each ovoid shaped barrel. The discovery of significant differences between these strains encourages further use of geometric morphometrics to identify genetic or transcript correlates of shape, either by classical trait QTL mapping (quantitative trait locus mapping) or expression QTL mapping [18,19]. Geometric morphometrics may also find use in describing the consequences of experimental genetic manipulations affecting the mouse cortical area map. Regardless of whether the cortical area map is studied from phenotype to gene or gene to phenotype, the analysis of cortical area map shape will help increase our understanding of cerebral cortical morphogenesis.

## Results

### Local differences: PMBSF

#### Procrustes superimposition

**Figure 3:**
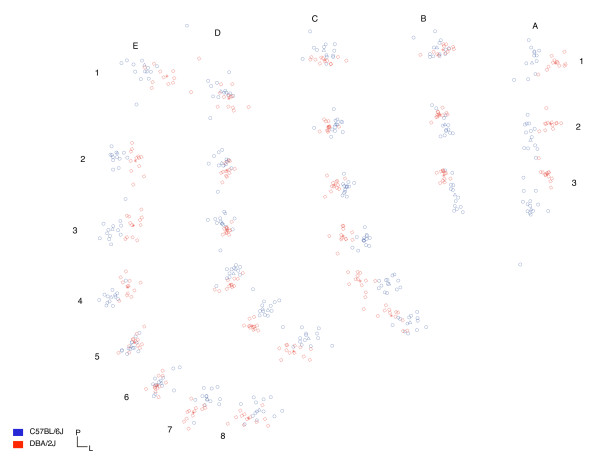
**PMBSF Procrustes scatterplot**. Procrustes superimposition of 26 barrel landmarks from each of 13 adult C57BL/6J mice and 12 adult DBA/2J mice. C57BL/6J is shown in blue circles. DBA/2J is shown in red squares. Blue triangles show C57BL/6J strain means. Red stars show DBA/2J strain means. Barrel identity is indicated as labeled. The figure shows the actual spread in the superimposed data, but also indicates the shape differences. One can readily discern strain differences in the position of any barrel by noting the relative separation by color for any given landmark point cloud. For example, in barrel A1, the red squares (DBA/2J) are lateral of the blue circles (C57BL/6J) and show no overlap. A similar lateral difference is seen in the point clouds for barrels A2 and A3, although the blue circles become progressively shifted anteriorly. See Figure [4] and text for a more complete description of noted shape changes. Figure [3] was composed in TwoGroup, a shape software title in the IMP series described in [9].

A Procrustes superimposition of 26 barrel landmarks from each of 13 adult C57BL/6J mice and 12 adult DBA/2J mice is shown in Figure 3. This superimposition procedure translates the starting configurations to a common origin, scales them to a common size, and rotates them to minimize an estimate of error (see Methods). The resulting differences represent changes in shape. Approximately 39% of variation in shape of the PMBSF is explained by strain. The probability that this shape conformation between strains could have arisen by chance was tested by permuting the data 10,000 times, and calculating for each permutation a statistic adapted for shape data, Goodall's F statistic. Goodall's F test compares the difference in mean shape between two samples relative to the shape variation found within the samples. One in 10,000 permuted data sets had a Goodall's F statistic as great or greater than the F = 15.0167 (df = 48, 1104) for the observed data, giving a significance probability of 0.0001.

**Figure 4:**
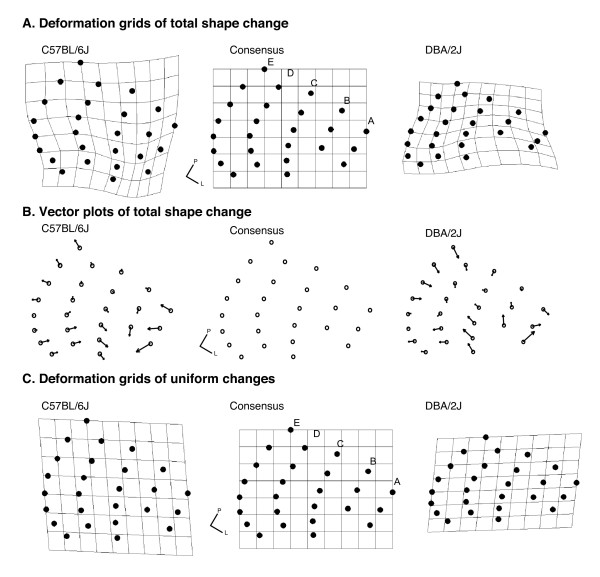
**PMBSF deformation grids and vector plots**. Deformation grids and vector plots of PMBSF landmarks. Whereas Figure 3 emphasizes the animal variation in shape grouped by strain, increased emphasis on the overall shape differences between strains is better illustrated by deformation grids and vector plots, two standard plot types for shape change depiction. 4A depicts total (uniform and non-uniform) shape differences in C57BL/6J, the consensus plot, and DBA/2J. The consensus (jargon of geometric morphometrics) represents the average, or midway interpolation, of the two strains. 4B shows vector plots of C57BL/6J and DBA/2J shapes, where the vector size is proportional to the observed changes. The noted shape changes in barrel row A mentioned in the caption for Figure 3 are more readily apparent in Figure 4A and 4B. See text for a more complete description of noted shape changes. 4C shows deformation grids of only the uniform effects, and so the grid lines are parallel. Effects in these plots have been increased 3-fold to better illustrate the differences between strains. The figures were composed in tpsRegr [32].

Figure 3 emphasizes the variation in the observed data, but differences in the position of barrels is also shown by using different colors and symbols for each strain. The majority of barrels in Figure 3 show visible separation by strain, with apparent patterning in the separations by barrel rows. In barrel row A in the PMBSF, DBA/2J barrel positions (red) are clearly offset laterally compared to C57BL/6J barrel positions (blue), with A2 and A3 becoming increasingly more anterior in C57BL/6J. Barrels B2 and B3 are also positioned more anterior in C57BL/6J, but the lateral separation as in row A is absent. In rows C and D there is modest separation of barrel positions by strain posteriorly (e.g. C1–3 and D1–3), with greater separation at the anterior ends of rows C and D (e.g. C4–6 and D4–6). In barrel row E, there appears to be the opposite effect, with more separation by strain apparent posteriorly (e.g., E1–4). Also, there appears to be mediolateral stretching and anteroposterior compression of the PMBSF in DBA/2J, relative to C57BL/6J. There is more space between barrel rows mediolaterally in DBA/2J relative to C57BL/6J, and less anteroposterior spread of barrel positions. These differences are more readily apparent in the deformation grids and vector plots in Figure 4, discussed below. Whereas Figure 3 emphasizes data spread by strain, the plots in Figure 4 are standard illustrations used to emphasize shape differences.

#### Deformation grids and vector plots

Thin-plate spline deformation grids for the Procrustes transformed barrel landmarks are shown for C57BL/6J, the consensus (average) of the two strains, and for DBA/2J (Figure 4A). Immediately noticeable from the general outline of the grids and spacing between barrel landmarks, is an anteroposterior expansion in C57BL/6J (taller grid) and the corresponding anteroposterior compression in DBA/2J (shorter grid). Differences in anteroposterior spacing between barrel landmarks is particularly apparent in barrel rows A and B. Less obvious, but still noticeable from the general grid outline, are mediolateral effects. The overall shape of the C57BL/6J grid in 4A is wider posteriorly, with the opposite effect apparent in DBA/2J – in other words, there is posterior lateral expansion and anterior lateral compression in the PMBSF of C57BL/6J. In addition to general anteroposterior and mediolateral differences in PMBSF shape, there are also focal anteroposterior and mediolateral effects. Along the A-P axis, there is straightening of the anterior ends of rows C, D, and E in DBA/2J (see lower left of the DBA/2J panel in 4A versus lower left of C57BL/6J). Along the M-L axis, rows A, B, and C are relatively compressed in C57BL/6J, while rows C, D, and E are relatively compressed in DBA/2J. Each of these general and focal effects described in the deformation grids can actually be discerned in the scatterplot, though it requires looking back and forth between the plots. Conversely, the comments made above for the scatterplot also are evident in the deformation grids. An alternative representation of the noted shape changes above is shown in 4B. These plots represent the change in barrel position by vectors.

#### Uniform components of shape

The plots in Figure 4A and 4B represent shape changes from the total variation shown in Figure 3. Shape variation can be decomposed into uniform and non-uniform components [20], and both the deformation grids and the vector plots in 4A or 4B illustrate the combined effects of uniform and non-uniform components of shape. Uniform components of shape do not bend the parallel grid lines in deformation plots such as those shown in Figure 4A. Two kinds of uniform shape differences are compression and shearing. Compression and shearing, for example, would make a rectangle more narrow or would make a rectangle into a parallelogram, respectively (see Methods for additional comments).

Figure 4C represents the uniform component of shape change for C57BL/6J and DBA/2J mean configurations. Regression of the partial warps for the X and Y uniform shape variables against the independent variables (here, strain) gives separate tests. For PMBSF, these test probabilities were 0.0005 and 0.0008, respectively. In addition to the anteroposterior compression noted above (the height of the DBA/2J parallelogram is less than in C57BL/6J), shearing is also evident in these plots (the longer diagonal of the DBA/2J plot lies on the A-P axis, whereas that of C57BL/6J lies on the M-L axis).

### Global differences: V1, S1, A1

#### Procrustes superimposition

**Figure 5:**
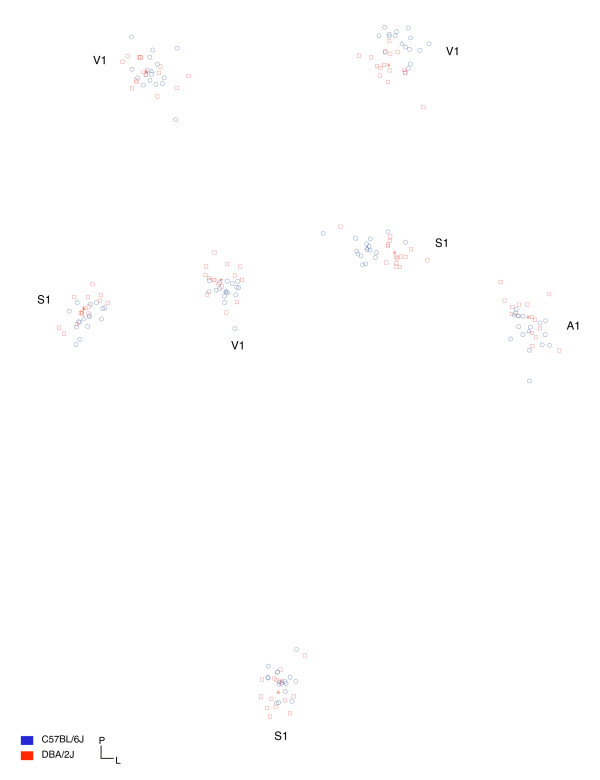
**V1, S1, and A1 Procrustes scatterplot**. Procrustes superimposition of 7 cortical landmarks from each of 16 adult C57BL/6J mice and f5 adult DBA/2J mice. C57BL/6J is shown in blue circles. DBA/2J is shown in red squares. Blue triangles show C57BL/6J strain means. Red stars show DBA/2J strain means. The figure shows the actual spread in the superimposed data, but also indicates the shape differences. One can readily discern strain differences in the position of any landmark by noting the relative separation by color for any given landmark point cloud. For example, the lateral posterior landmark for V1 and for S1 show little overlap between blue circles and red squares. See Figure 6 and text for a more complete description of noted shape changes. The figure was composed in TwoGroup [9].

A Procrustes superimposition of 7 landmarks across V1, S1, and A1 from each of 16 adult C57BL/6J mice and 15 adult DBA/2J mice is shown in Figure 5. Approximately 15.2% of variation in the shape of the cortical area map is explained by strain. Using a permutation test to confirm strain differences in shape variation, 18 in 10,000 permuted data sets had a Goodall's F statistic as great or greater than the F = 5.1993 (df = 10, 290) for the observed data, giving a significance probability of 0.0018.

Separation by strain is evident in the lateral and rostral V1 landmarks, and the most lateral S1 landmark. Other landmarks show no clear separation between strains.

#### Deformation grids and vector plots

**Figure 6:**
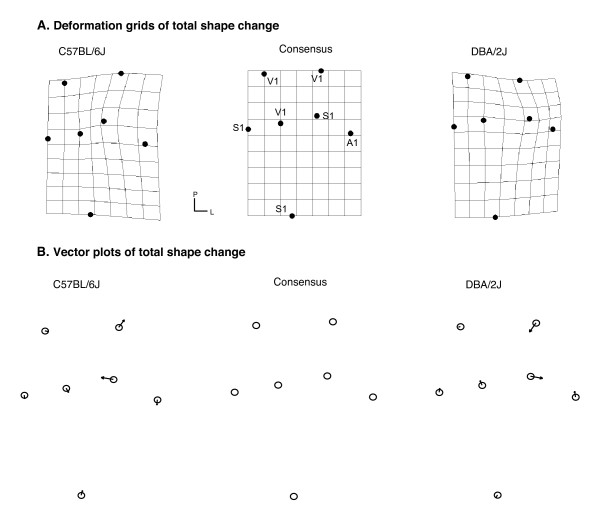
**V1, S1, and A1 deformation grids and vector plots**. Deformation grids and vector plots of the primary sensory cortex landmarks. Whereas Figure 5 emphasizes the animal variation in shape grouped by strain, increased emphasis on the overall shape differences between strains is better illustrated by deformation grids and vector plots, two standard plot types for shape change depiction. 6A shows deformation grids for C57BL/6J, the consensus, and DBA/2J. 6B shows vector plots for C57BL/6J and DBA/2J, where the vector size is proportional to the observed changes. Effects in these plots have been increased 3-fold to better illustrate the differences between strains in the raw scale scatterplots above. See text for a more complete description of noted shape changes. The figures were composed in tpsRegr [32].

Thin-plate spline deformation grids for the Procrustes transformed primary sensory cortex landmarks are shown in Figure 6A for C57BL/6J, the consensus or average, and DBA/2J. Figure 6B shows the corresponding vector plots. The positions of the lateral S1 landmark and the A1 landmark are in closer proximity in DBA/2J relative to C57BL/6J. This may reflect local changes in the PMBSF already described. Also in Figure 6A–B, V1 is anteriorly and laterally expanded in C57BL/6J relative to DBA/2J.

#### Uniform components of shape

Despite the presence of significant uniform shape components in the local PMBSF, and the presence of significant shape differences across V1, S1, and A1, there were no significant uniform shape components in this second landmark configuration.

#### V1 subset

The 3 landmarks for the triangular shape of V1 were analyzed separately. Approximately 29.7% of variation in shape of V1 is explained by strain. Using a permutation test to confirm strain differences in shape variation, 1 in 10,000 permuted data sets had a Goodall's F statistic as great or greater than the F = 12.3117 (df = 2, 58) for the observed data, giving a significance probability of 0.0001. V1 appears elongated both in the anteroposterior and mediolateral dimensions in C57BL/6J relative to DBA/2J.

## Discussion

**Figure 7:**
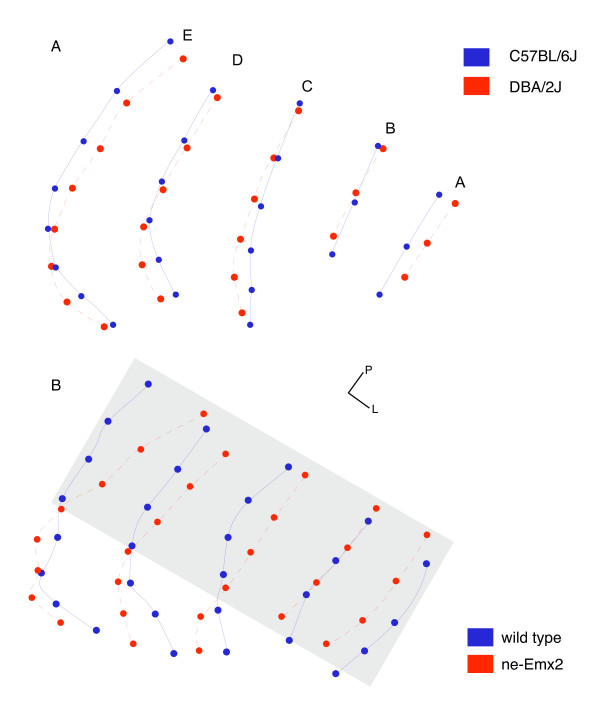
**PMBSF Procrustes superimposition in *ne-Emx2 *mice**. (7A) Procrustes superimposition of 26 barrel landmarks from C57BL/6J (blue) and DBA/2J (red) emphasizing the strain means from Figure 3 above. (7B) Procrustes superimposition of barrels from one wild-type mouse (blue) and one *ne-Emx2 *mouse (red) from figure 3 of [5] (original figure provided by D.D. O'Leary). In 7B we note two main shape differences. The first is highlighted by the gray box. Wild-type PMBSF shows lateral stretching relative to *ne-Emx2 *mice for the posterior parts of barrel rows A to E. Second, in the area not highlighted by the gray box (anterior ends of rows C, D, and E), there is rostral stretching in *ne-Emx2 *mice. This aspect is similar to the effect seen in DBA/2J versus C57BL/6J.

Using a novel application of established methods of geometric morphometrics for the analysis of shape, we have demonstrated differences in the primary sensory areas of cortex between the inbred, isogenic mouse strains C57BL/6J and DBA/2J. We found significant differences in the shape of the posterior medial barrel subfield using the positions of the barrels as landmarks, and we found additional differences between minimal landmarks of V1, S1, and A1. Combined with a prior study of planar areal differences in C57BL/6J and DBA/2J [6], the cortical area maps of these common, inbred mouse strains have now been shown to differ significantly in measures of both primary sensory cortex area and shape. Rostrolateral differences between C57BL/6J and DBA/2J in the PMBSF and in V1 are reminiscent of effects seen in mice over expressing the transcription factor Emx2 (Figure 1 and [5]). Closer inspection of the Procrustes superimposition of barrels between inbred strains and the superimposition between transgenic *ne-Emx2 *mice reveals interesting parallels but differences as well (Figure 7). While we do not claim the phenotypic differences between C57BL/6J and DBA/2J shown here are evidence of differences in *Emx2 *expression across these strains, the described shape differences could nonetheless result from polymorphism in early cortical patterning genes or differences in gradients or local enrichments of transcription factors, including Emx2.

Cortical arealization initiates from early secreted ligands and graded distribution of transcription factors, which trigger the activation of distinctive area-specific morphogenetic programs, and result in an ordered topology of species-typical primary sensory and motor cortical areas (see [1-4] for review). Manipulation of transcription factor gradients have altered caudal and rostral cortical allotment to specific cortical fields. For example, Hamasaki et al., [5] caused a caudal over expression of *Emx2*. Homzygote *ne-Emx2 *mice, compared to wild-type mice, showed a gradient of change in cortical area size from the caudal to the rostral poles. Transgenic *ne-Emx2 *mice showed a 52% increase in V1, a 25% decrease in PMBSF, and a 36% decrease in motor cortex. There was also a rostrolateral shift in the positions of S1 and A1. Hamasaki et al., [5] have been the only group to quantitate in some detail the anatomical effects of their genetic manipulations, although their methods are unrelated to the geometric morphometric descriptions of shape in two dimensions used in the present paper.

The application of shape statistics to sections of the flattened cortical sheet is an obvious but powerful innovation that has the potential to improve and unify attempts to detect and describe effects of genetic manipulations on cortical arealization. In addition to improving phenotypic description of genetic manipulations putatively affecting cortex arealization, shape analysis of the cortical area map enables forward genetic efforts to isolate quantitative trait loci (QTLs). This approach would correlate measured quantitative phenotypic variation in cortex to genetic variation between mice or lines of mice (e.g., [21,18]). Shape statistics has been applied successfully to isolate a large number of QTLs for mouse mandible shape [22]. Cortical morphogenesis has been predominantly studied using experimental (reverse) genetic strategies. Relatively few genes have been identified that play a role in specifying the cortical area map, and our understanding of the genetic network active during cortical specification is poor. Genes that have been identified, however, are well supported [2,4,1,3]. Forward genetic approaches to cortical morphogenesis would provide a complementary strategy. Although the conversion of QTLs to identified genes is difficult, advances in forward genetic methods have demonstrated success [23-25].

Another approach is to use gene microarray technologies to identify spatial or temporal differences in gene expression in the developing cortex. Use of microarrays in genetically manipulated mice [26], or mice of differing genetic backgrounds [26,27], is a promising discovery approach to understanding the network of transcription factors that must coordinately define the cortical area map. Sansom et al., [26], used microarrays to characterize the early cortical transcriptome of mice lacking a receptor important in Fgf signaling, and identified and validated the novel target gene *Mest*. Given differences in V1 area and shape between C57BL/6J and DBA/2J strains, microarrays could be used to test for differences between strains in the transcriptome from caudal, embryonic cortex. Although the work of Sansom et al., [26], is a model study, it lacked direct correlation of the transcriptome differences to cortical map phenotypes.

An approach that integrates genetic, transcript, and phenotypic variation would use the genetic reference population of BXD recombinant inbred lines [28]. This panel of genetically varied lines derived from C57BL/6J and DBA/2J strains can be phenotyped for cortical area and shape differences (as in [6] and this paper) and also characterized for anterior-posterior early cortical gene expression differences between lines (as in [26] or [27]). Since both parental inbred strains are fully sequenced, the full complement of genetic variation is archival in the BXD and need not be re-genotyped (see [29]). Phenotype to genotype (classical trait QTLs), phenotype to transcript, and transcript to genotype (expression QTLs) relationships could then all be evaluated, genome-wide [19]. Because we have now shown that the parental strains C57BL/6J and DBA/2J markedly differ in both quantitative measures of cortex area size [6] and shape, this assures variation in the derivative BXD lines, and provides an empirical basis for using the BXD panel to study cortical development.

## Conclusion

C57BL/6J and DBA/2J have markedly different cortical area maps, in both size and shape. These differences suggest polymorphism in genetic factors underlying cortical specification, even between common isogenic strains. Comparing cortical phenotypes between normally varying inbred mice or between genetically modified mice can identify genetic contributions to cortical specification. Geometric morphometrics represents an additional quantitative tool for the study of cortical development, regardless of whether it is studied from phenotype to gene or gene to phenotype.

## Methods

### Animals

Mice were purchased from Jackson Laboratories at 4–6 weeks of age, housed on a 12:12 light:dark cycle in same sex groups in standard laboratory animal cages (5 animals per cage). All experimental procedures were performed in accordance with the *Guidelines for the Care and Use of Laboratory Animals *published by the National Institutes of Health (publication 86–23) and the Vanderbilt University Animal Care and Use Committee. Mice were provided a standardized diet and clean water ad libitum. Mice at this age are not compromised by known visual and or auditory sensorineural deficits common to older animals of these strains. None of the mice used had visible body or facial wounds.

### Tissue

At 6–8 weeks of age (14 day age span), young adult mice were brought to complete anesthesia with a sodium pentobarbital overdose (100 mg/kg) injected intraperitoneally (IP), and then transcardially perfused with 0.1 M phosphate buffered 0.9% saline wash followed by 3% buffered paraformaldehyde fixative. Intact brains were removed from the skull, and the cortex was dissected free of the underlying white matter. Dissected cortices were flattened between glass slides and transferred to 30% sucrose for 12–18 hours. Cortices were sectioned parallel to the cortical surface at a thickness of 70–80 *μ*m on a freezing, sliding microtome, stained for cytochrome oxidase according to the method of Wong-Riley [30], mounted on glass slides, air dried, and coverslipped. Thicker sections encourage all cortical areas to be within a single section, and are less prone to deformation or tearing.

### Landmarks

A scale bar and the outlines of cortical regions of interest (ROIs) were drawn under a light microscope with a camera lucida attachment. Regions of interest included neocortex (C), visual cortex (V1), auditory cortex (A1), somatosensory cortex (S1), and select barrels of the posterior medial barrel subfield (PMBSF). Barrels drawn in rows A, B, C, D, and E were standardized to 5, 4, 6, 7, and 8 barrels, respectively (alpha, beta, gamma, and delta barrels were also included). Digital scans of the drawings were imported into a computer and landmark coordinates acquired with NIH ImageJ software after scale bar calibration [31]. All data were acquired blind to animal and strain identity, and the order of acquisition was independent of animal or strain.

Two landmark configurations were generated for this study, the first to assess local shape differences in the PMBSF, the second to assess global shape differences across the cortical sheet (see Figure 2). Landmarks are points that can be located precisely (repeatedly) on the anatomical structure under study and display a one-to-one correspondence among the specimens included in the study. The first landmark configuration consisted of 34 barrel centroids. Centroids are the average coordinates of a set of coordinates. In ImageJ a centroid is the average X and Y from all pixel coordinates within a region of interest. Due to missing data in some animals for barrels alpha, beta, gamma, delta, A4–A5, B4, and D7, the final data set consisted of 26 landmarks for barrels A1–A3, B1–B3, C1–C6, D1–D6, and E1–E8, in 13 C57BL/6J mice (4 males, 9 females) and 12 DBA/2J mice (8 males, 4 females). The methods of shape analysis used in this paper required that all landmarks be present in all cortices measured. The second landmark configuration consisted of 7 landmarks, 3 that reduced V1 to a triangle, 3 that reduced S1 to a triangle, and 1 that was the centroid of A1. For this configuration, 16 C57BL/6J mice (6 males, 10 females) were compare to 15 DBA/2J mice (9 males, 6 females). In this paper sex effects in the cortical area maps were not a focus, and sex was determined not to be a significant factor by Goodall's F tests performed separately on each strain for each landmark configuration (each P > 0.05). Li et al., [14], also recently looked at the barrels of C57BL/6J, DBA/2J, and a small sample of derivative BXD lines, and found no sex differences in PMBSF area, although shape was not examined. Li et al., also found no differences in PMBSF area over a 21 day age spanning the same ages sampled in this study. In our data, over a 14 day age span, we found no significant difference that we could attribute to age, in either strain for either landmark configuration (Goodall's F tests, each P > 0.05). No substantive differences in equipment or procedures confounded the significant strain differences we report in this paper.

Reliability of landmark configurations was assessed by acquiring (drawing) each landmark configuration in triplicate for each animal. The first set of drawings was discarded to avoid training effects. The second and third sets were tested for between set differences, using shape analysis. None were found for either landmark configuration (Goodall's F tests, P > 0.05). The final data set used for shape analysis was formed by averaging over the second and third drawing sets, as well as hemisphere within animal, if two hemispheres were drawn. If two hemispheres were drawn, they were appropriately flipped to the same orientation. In this paper we do not consider average differences in left and right hemispheric cortical maps. Studies in rats [15] and mice [14] found no left and right hemispheric differences in PMBSF area, although shape was not examined. Before shape analysis, final data sets were preanalyzed by the program tpsSmall [32] to confirm variation was appropriate (small enough) for shape analysis.

### Shape

The collection of complex multivariate statistical methods used to analyze shape are organized around Kendall's definition of shape [33]. Shape is defined as all the geometric information that remains when location, scale and rotational effects are filtered out from an object or landmark configuration. Shape analysis in this study made use of generalized Procrustes analysis (GPA) to superimpose landmark configurations and remove nonshape variation in the landmark coordinates [34,20,35]. The algorithm of this method involves three transformations [7,9]. First, the centroid of each configuration is translated to the origin by subtracting centroid coordinates from each landmark. Second, the configuration is then scaled by dividing each landmark coordinate by the centroid size of that configuration. The centroid size is the square root of the summed squared distances of each landmark from the centroid of the landmark configuration. Third, with respect to a given configuration, another configuration is rotated to minimize the summed squared distances between labeled landmarks. In tpsRegr, this is done iteratively: one reference configuration is chosen and every other configuration is fitted to it, but from the second round on, the average of the coordinates in the previous fit is used as a reference. Final superimposition is not influenced by which landmark configuration is chosen to begin the procedure.

**Figure 8:**
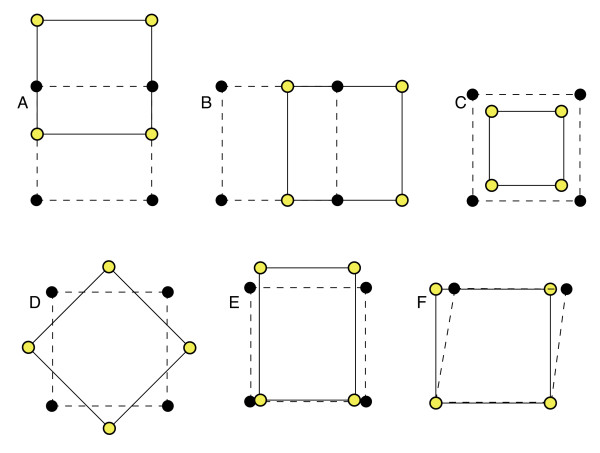
**Uniform transformations**. There are six uniform transformations. Translation in X (8A) or in Y (8B), scaling (8C), and rotation (8D) do not alter shape, and are removed prior to shape analysis. Translation, scaling, and rotation are not components of shape change, because shape is by definition invariant to these transformations. Compression (8E) and shearing (8F) do alter shape, but are different from non-uniform shape components. Reprinted from [9], page 136, with permission from Elsevier.

In the analysis of shape, there are both uniform and non-uniform components to shape variance. When considering an elastic sheet of graph paper, uniform changes are those that leave parallel lines parallel. Non-uniform changes bend lines. Figure 8 shows two kinds of uniform changes, compression and shearing (E-F), that alter shape. Translation, scaling, and rotation (A-D) are not components of shape change, because shape is by definition invariant to these transformations. Uniform components of shape variation can be computed separately [20] and represented separately. It is not clear if separating the uniform shape component is helpful or more interpretable for a given biological question. Considering that the cortical area map is thought to be specified by overlapping early gradients or local enrichment of secreted ligands or gradually expressed transcription factors [3], a combination of uniform and non-uniform shape changes might be predicted. Hamasaki et al., [5] increased the slope of a rostrocaudal gradient of the transcription factor Emx2 in the cortex of transgenic mice relative to wild-type mice. Phenotypically, transgenic caudal cortical areas were increased in size and shifted anteriorly, at the expense of rostral cortical areas, but the total cortical sheet was unchanged in size. An over-simplified analogy might be a ladder of fixed length, where the rung distance decreases from the bottom of the ladder to the top, and landmarks are where the rungs are bolted to the rails. Although geometric morphometrics provides methods to describe and disentangle both uniform and non-uniform components of cortical area map shape, in both *ne-Emx2 *mice and the C57BL/6J and DBA/2J inbred strains, shape seems best characterized by the total shape variation.

A statistical test adapted to the coordinates produced by Procrustes fit is Goodall's F test [36], which compares the difference in mean shape between two samples relative to the shape variation found within the samples. To determine statistical significance we employed a permutation test based on Goodall's F statistic. In this test, the data are permuted (randomized) and Goodall's F statistic is calculated. This is done 10,000 times. The proportion of Goodall's F statistics from permuted data sets as great or greater than the Goodall's statistic on the original data set is given as the significance probability. Use of permutation relaxes some of the restrictive assumptions of Goodall's F test. Goodall's F test only considers the total amount of shape variation, and does not consider the directionality of the variation. With small samples (relative to the number of landmark coordinates), as in this study, this is a useful property. Indeed, the sample size in this study is too small to use alternative MANOVA multivariate statistics to test for shape differences in the PMBSF, given the number of barrel landmarks and the number of mice measured.

In addition to scatter plots of Procrustes transformed landmark data, broken down by symbol to represent strain (e.g., Figure 3 above), thin-plate spline deformation grid plots are used (e.g., Figure 4 above). A thin-plate spline deformation grid represents a smooth interpolation, mapping the coordinates of one landmark configuration into another. These plots are analogous to an elastic sheet of graph paper that is stretched to map one morph to another, and are in the spirit of the historically famous efforts of D'Arcy W. Thompson [37]. Deformation grids are useful for visualizing changes between landmarks over an entire configuration. Aside from deformation grids, vector plots are also provided. These represent the change in landmark positions by a vector with size proportional to the observed differences.

Although the statistical methods used in this paper are complex, reliable software for geometric morphometrics is readily and freely available and makes these methods accessible to neuroscientists. The primary software used in this study was tpsRegr by statistician F. James Rohlf [32]. The strain differences reported above were confirmed using two additional software titles that implement Procrustes fit and Goodall's F test: TwoGroup by H. D. Sheets [9], and shapes, an R package by Ian Dryden [7].

## Authors' contributions

DCA and CEC conceived and designed the study. DCA, CEC, FW, and MG collected data. DCA analyzed the data. DCA and CEC prepared the manuscript. All authors have read and approved the final manuscript.
